# Capsaicin, the Spicy Ingredient of Chili Peppers: Effects on Gastrointestinal Tract and Composition of Gut Microbiota at Various Dosages

**DOI:** 10.3390/foods11050686

**Published:** 2022-02-25

**Authors:** Qunran Xiang, Xin Tang, Shumao Cui, Qiuxiang Zhang, Xiaoming Liu, Jianxin Zhao, Hao Zhang, Bingyong Mao, Wei Chen

**Affiliations:** 1 State Key Laboratory of Food Science and Technology, Jiangnan University, Wuxi 214122, China; xiangqunran@foxmail.com (Q.X.); xintang@jiangnan.edu.cn (X.T.); cuishumao@jiangnan.edu.cn (S.C.); zhangqx@jiangnan.edu.cn (Q.Z.); liuxm@jiangnan.edu.cn (X.L.); zhaojianxin@jiangnan.edu.cn (J.Z.); zhanghao61@jiangnan.edu.cn (H.Z.); chenwei66@jiangnan.edu.cn (W.C.); 2 School of Food Science and Technology, Jiangnan University, Wuxi 214122, China; 3 National Engineering Research Center for Functional Food, Jiangnan University, Wuxi 214122, China

**Keywords:** capsaicin, gastrointestinal, inflammation, neuropeptides, TRPV1, gut microbiota

## Abstract

Capsaicin (CAP) is an ingredient of peppers that has biological activities at low doses but causes gastrointestinal (GI) discomfort at high doses. However, the GI effects of high doses of CAP and the evaluation criteria to determine this remain unknown. To elucidate the dose-related effects of CAP on GI health, CAP was administered to mice at 40, 60, and 80 mg/kg doses. The results showed that 40 mg/kg CAP did not negatively affect GI tissues, while 60 and 80 mg/kg CAP damaged GI tissues and caused significant inflammation in the jejunum, ileum, and colon. The levels of serum substance P (SP) and calcitonin gene-related peptide (CGRP) were CAP-dose-dependent, and short-chain fatty acids (SCFAs) content significantly increased in the 80 mg/kg group. Correlation analysis revealed that the underlying mechanisms might be related to the regulation of gut microbiota, especially *Bifidobacterium*, *Lactobacillus*, *Faecalibacterium*, and *Butyricimonas*. These results suggest that oral administration of 60 and 80 mg/kg CAP in mice causes intestinal inflammation and high levels of serum neuropeptides and cecal SCFAs, which may be related to alterations in gut microbiota.

## 1. Introduction

Capsaicin (CAP) is the main pungent ingredient of chili peppers [1], which are spices with a unique spicy flavor widely consumed in various diets. CAP has demonstrated broad potent biological characteristics, including antioxidant, anti-obesity, pain-alleviating, and anti-inflammation effects [2]. Nevertheless, recent studies have revealed that CAP shows bioactivities under low doses but tends to have side effects under high doses. When CAP is continuously consumed at high levels, people may feel some uncomfortable gastrointestinal (GI) symptoms—such as heartburn, diarrhea, pain, and other symptoms—in their daily life [3]. Therefore, the GI effects of CAP ingestion have received increasing attention.

Owing to its pungency, high-dose CAP may inhibit gastric acid production [4], damage the GI mucosa by inducing gastric inflammation [5,6], cause structural changes of the intestinal barrier [7,8,9], and further result in other GI symptoms [10,11,12]. Nevertheless, results from studies on CAP vary because of the dosage and duration of CAP and the species and characterization of research subjects, making it difficult to confirm the specific GI effects of CAP. Therefore, there are no uniform and clear experimental models and evaluation criteria to determine the effects of CAP ingestion on GI health.

Against this background, the present research aimed to elucidate the effects of CAP on GI health and to investigate the mechanisms of CAP-induced GI injury. Tissue inflammation, histopathological injury, and serum levels of GI neuropeptides in mice were detected to reflect the effects of different oral doses of CAP on GI health. Furthermore, a correlation analysis was performed to determine if CAP regulates short-chain fatty acids (SCFAs) and the composition of gut microbiota. This may correlate CAP ingestion with GI health and provide more insight into biological safety studies on CAP.

## 2. Materials and Methods

### 2.1. Animals and Experimental Design

Twenty-four SPF male C57BL/6J mice (18 ± 2 g, 6-week-old) were purchased from Charles River (Beijing, China) and reared at a temperature of 25 ± 2 °C under an artificial lighting period (12 h light/12 h dark) in a barrier facility of the Animal Center of Jiangnan University. After seven days’ adaptive feeding, all mice were randomly divided into four groups of six mice each.

The experimental protocol is presented in Table 1. Briefly, the control group was intragastrically administered 200 μL of vehicle (3% ethanol and 10% Tween 80 in 0.9% saline) daily [13,14], while three CAP groups were intragastrically administered 200 μL of different concentrations of CAP dissolved in vehicle (Sinopharm Chemical Reagent Co., Ltd., Shanghai, China). The intragastrically administrated CAP concentrations were 40, 60, and 80 mg/kg. During the seven-day trial, the bodyweight, food intake, and water intake were counted and recorded every day. After seven days, the mice were anesthetized and sacrificed. Blood samples were collected from the mice orbital plexus and then centrifuged at 3000× *g* for 30 min to get the serum. Fresh fecal and cecal content were collected, then the samples were immediately frozen at −80 °C. Tissues of the stomach, jejunum, ileum, and colon were collected, and a portion of each tissue was separated and fixed with 4% paraformaldehyde, the rest was frozen at −80 °C. All animal experiments used in this study were approved by the Ethics Committee of Jiangnan University, China (JN. no. 20200630c0640815(126)) and complied with the guidelines of the 2010/63/European Community.

### 2.2. Histopathology and Measurement of Inflammatory Cytokine Levels in GI Tissues

Some tissues of the stomach, jejunum, ileum, and colon were fixed in 4% paraformaldehyde and stained with hematoxylin and eosin (H&E). Images of the paraffin sections were taken by scanning with a software named Pannoramic MIDI Digital Slide Scanner (3D-Histech Co., Ltd., Budapest, Hungary). The software CaseViewer 2.3 and Image J were used to measure the histopathological injury, and the valuation criteria are referred to the previous study [15].

Tissues, including the remaining stomach, jejunum, ileum, and colon, were homogenized with the phosphate buffer (containing 1% protease inhibitor cocktail) and centrifuged at 8000× *g* for 15 min. The concentrations of interleukin (IL)-10, TNF-α, and IL-1β were quantized using the ELISA kits (R&D Systems, Minneapolis, MN, USA).

### 2.3. Measurement of Serum Neuropeptide

The contents of substance P (SP) and calcitonin gene-related peptide (CGRP) in the serum of the mice were analyzed using commercial ELISA kits (Mlbio, Shanghai, China).

### 2.4. Determination of Levels of SCFAs in Cecal Content

The contents of SCFAS in the ceca were analyzed using gas chromatography–mass spectrometry (GC-MS) as previously described [16], including acetic, propionic, and butyric acid. The cecal contents were freeze-dried and approximately 50 mg of the samples were suspended with saturated NaCl for 30 min. SCFAs were then extracted by diethyl ether and then quantified by GC-MS (QP2010 Ultra system, Shimadzu Corporation, Kyoto, Japan).

### 2.5. 16S rRNA Gene Sequencing

Total DNA of fecal samples was extracted by a commercial kit (MP Biomedicals; Carlsbad, CA, USA). The 16S rRNA gene was PCR-amplified with primers (341F/806R) specific for the V3–V4 region. The PCR products were stained with 4% nucleic acid dye, electrophoresed on a 1.5% agarose gel (SBS Genetech Co., Ltd., Beijing, China), and used a QIAquick Gel Extraction Kit (Qiagen GmbH, Hilden, Germany) to purify. The purified DNA concentration was determined using Qubit 4 Fluorometer (Life Technologies Corporation, Carlsbad, CA, USA) and the amplicon libraries were pooled in equimolar amounts and sequenced by MiSeq (Illumina, Santiago, CA, USA) at Jiangnan University (Wuxi, China). The sequence data were mapped into operational taxonomic units (OTUs) with a similarity of 97%.

### 2.6. Statistical Analysis

Statistical analyses were conducted with Origin 2018 and SPSS v22.0. All data are indicated as the mean ± standard error (SEM), and one-way analysis of variance (ANOVA) according to LSD post hoc tests was used to compare multiple groups. *p* < 0.05 was considered as significant. Microbiota analysis was performed using QIIME 2 pipeline, Shannon indexes, and Chao1 index, while principal coordinates analysis (PCoA) was implemented using marker data profiling (https://www.microbiomeanalyst.ca/ (accessed on 7 August 2021)). Linear discriminant analysis (LDA) effect size (LEfSe) was implemented online (http://huttenhower.sph.harvard.edu/galaxy (accessed on 8 August 2021)). Correlation analysis was implemented using R version 3.6.3.

## 3. Results

### 3.1. Effects of CAP on Body Weight and Food and Water Intake

**Figure 1:**
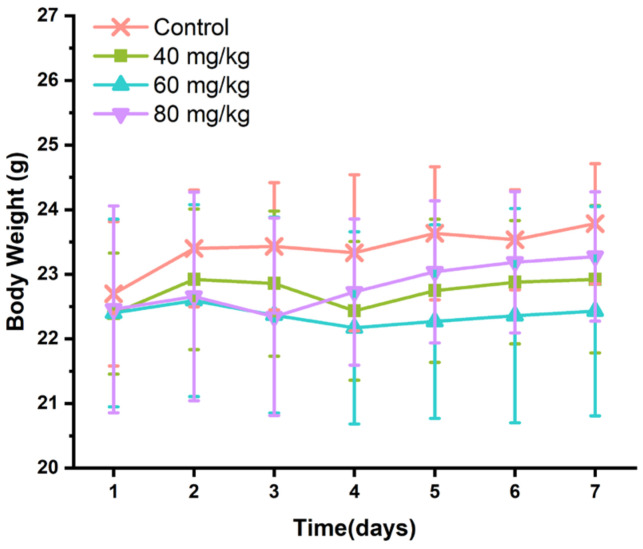
Effects of CAP on body weight of mice.

With the daily bodyweight of the mice measured, no significant weight change was observed in the 7-day treatment with CAP (*p* > 0.05). Interestingly, the average bodyweight of mice in different doses of CAP groups was lower than that in the control group, but not significant (Figure 1). Meanwhile, there were no significant differences in food intake and water intake (Supplementary Figure S1) between the experimental groups, but the mice treated with high-dose CAP (60 and 80 mg/kg) had a more erratic and dispersive water intake than the control group.

### 3.2. Effects of CAP Administration on the Histopathology of GI Tissues

**Figure 2:**
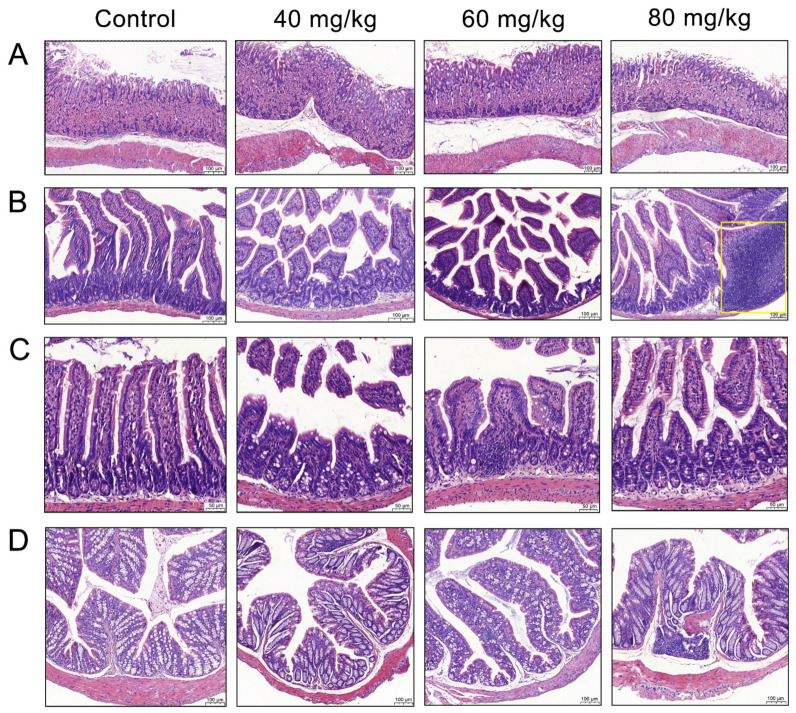
Morphology of the different gastrointestinal tissues. (**A**) Stomach, (**B**) jejunum, (**C**) ileum, and (**D**) colon.

Owing to the pungent properties of CAP, the morphology of GI tissue was observed to determine whether CAP caused histopathological injury. H&E staining was used for the histopathological evaluation of the stomach, jejunum, ileum, and colon (Figure 2A). 

**Figure 3:**
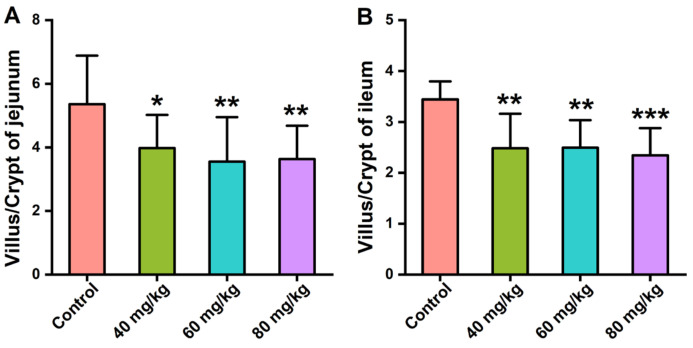
Effects of CAP on the villi/crypt of the intestine, specifically in the (**A**) jejunum and (**B**) ileum. *p* < 0.05, ** *p* < 0.01, and *** *p* < 0.001 vs. control.

The gastric mucosa of the control group was intact, and their nuclei were clearly visible. The groups treated with different concentrations of CAP showed slight cell vacuolization, but there was no significant difference when compared with the control group. The jejunum of the control group mice had tidy villi with healthy crypt structures and intact mucous membranes, while the jejunal villi in the CAP-treated groups were irregularly arranged with local villus breakage and shedding (Figure 2B). The group treated with 80 mg/kg CAP showed inflammatory cell infiltration. We further evaluated the ratio of villus height to crypt depth (V/C) in the jejunum. Compared with the control group, treatment with 40, 60, and 80 mg/kg CAP reduced the V/C ratio in the jejunum by 25.7%, 33.7%, and 32.2%, respectively (Figure 3A). Similar results were observed in the histopathology of the ileum, where the ileal villi of the CAP-treated groups were shortened. Treatment with 40, 60, and 80 mg/kg CAP reduced the V/C ratio of the ileum by 27.9%, 27.6%, and 31.9%, respectively, compared with the control group (Figure 3B), accompanied by local villus breakage and crypt hyperplasia (Figure 2C).

The colon tissue of mice in the control group showed enriched goblet cells and healthy crypt structures with tidy villi, while the mice in the CAP-treated groups showed inflammatory cell infiltration, and loss of crypt and goblet cells (Figure 2D). Remarkably, mice treated with 60 mg/kg CAP showed a loss of mucus-producing goblet cells compared with mice in the control group, and inflammatory cell infiltration was evident in the 80 mg/kg CAP-treated mice. The statistical analyses of the colonic crypt height and goblet cell numbers were shown in Supplementary Figure S2.

### 3.3. Effects of CAP on the Levels of Inflammatory Cytokines in Gastrointestinal Tissues

**Figure 4:**
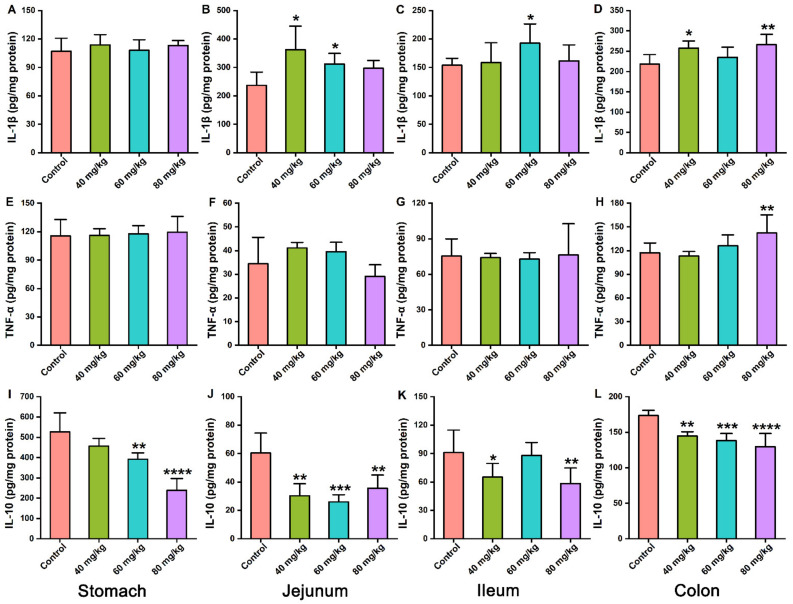
Effects of CAP on inflammatory cytokine levels in the stomach, jejunum, ileum, and colon tissues. (**A**–**D**) IL-1β, (**E**–**H**) TNF-α, and (**I**–**L**) IL-10. * *p* < 0.05, ** *p* < 0.01, *** *p* < 0.001, and **** *p* < 0.0001 vs. control.

Levels of inflammatory cytokines in gastrointestinal tissues were measured to evaluate whether CAP induced gastrointestinal injury via an inflammatory effect. The results revealed that CAP administered orally at a dose of 40–80 mg/kg increased IL-1β levels in the jejunum and colon (Figure 4B,D) compared with the control group. The levels of TNF-α in the stomach, jejunum, ileum, and colon showed no significant differences among all CAP-treated groups, except in the colon of the 80 mg/kg CAP group (Figure 4E–H). However, all gastrointestinal tissues showed a decrease in IL-10 levels after CAP treatment (Figure 4I–L). Remarkably, the decrease seemed to be CAP-dose-dependent in colon tissues (Figure 4L). Consequently, oral administration of CAP influenced the levels of anti-inflammatory cytokines in the stomach and ileum, but did not induce severe inflammatory injury. However, CAP caused inflammatory injury in the jejunum and colon at high doses (80 mg/kg) by increasing IL-1β and TNF-α levels and decreasing IL-10 levels.

### 3.4. Effects of CAP on the Neuropeptide Levels in Serum

**Figure 5:**
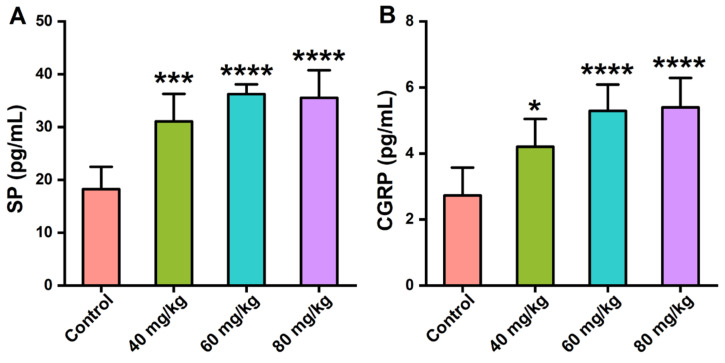
Effects of CAP on the serum levels of gastrointestinal neuropeptides. (**A**) SP levels and (**B**) CGRP levels. * *p* < 0.05, *** *p* < 0.001, and **** *p* < 0.0001 vs. control.

The serum levels of SP and CGRP were measured to reflect the effects of CAP on the secretion of gastrointestinal neuropeptides in mice. As shown in Figure 5, CAP orally administered at doses of 40, 60, and 80 mg/kg significantly increased the serum levels of SP and CGRP, which were CAP-dose-dependent.

### 3.5. Effects of CAP on the Levels of Cecal SCFAs

**Figure 6:**
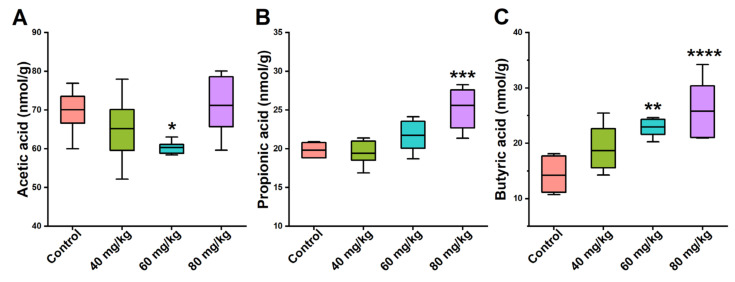
Effects of CAP on the levels of cecal SCFAs. (**A**) Acetic acid, (**B**) propionic acid, and (**C**) butyric acid. * *p* < 0.05, ** *p* < 0.01, *** *p* < 0.001, and **** *p* < 0.0001 vs. control.

SCFAs are the main metabolites of intestinal microorganisms, mainly due to the breakdown of polysaccharides or dietary fiber. Therefore, the levels of SCFAs in cecal content—including acetic, propionic, and butyric acid—in the mice were quantified using GC-MS. Compared with the control group, the cecal level of acetic acid in the 60 mg/kg CAP-treated group was dramatically lower, while all the other groups showed no significant difference (Figure 6A). Orally administered CAP caused a dose-dependent increase in the cecal level of propionic acid. However, compared with the control group, only the 80 mg/kg dose showed a significant difference (Figure 6B). The trends in the levels of butyric acid in the 40, 60, and 80 mg/kg CAP-treated mice were similar to those of propionic acid. After 7-day treatment with 60 and 80 mg/kg CAP, butyric acid levels significantly increased (Figure 6C).

### 3.6. CAP Modulates the Composition of Gut Microbiota

**Figure 7:**
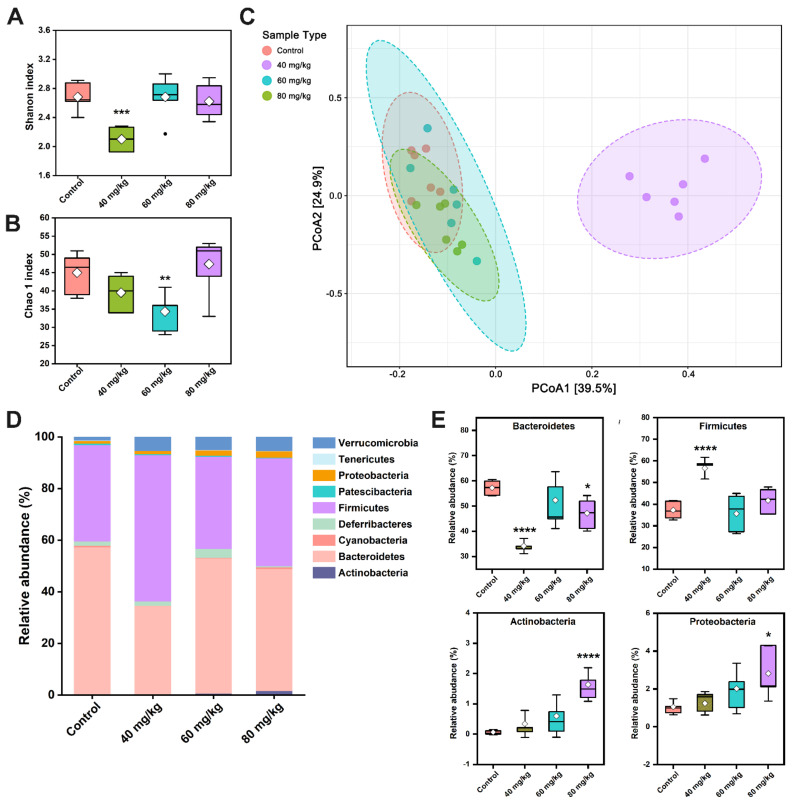
Effect of CAP on the overall structure of gut microbiota. The (**A**) Shannon index, (**B**) Chao1 index, and (**C**) PCoA, with extended functionality for the labeling groups and with normal probability ellipsoids for different groups. (**D**) Distribution of bacterial taxa at the phylum level. (**E**) Comparison of the main phyla in the different groups: Bacteroidetes, Firmicutes, Actinobacteria, and Proteobacteria. * *p* < 0.05, ** *p* < 0.01, *** *p* < 0.001, and **** *p* < 0.0001 vs. control.

To assess if various doses of CAP regulate the gut microbiota, high-throughput sequencing was performed for analysis and comparison. The alpha diversity is reflected by the Shannon index and Chao1 index. Compared with the control group, the 40 mg/kg CAP-treated group showed dramatically decreased Shannon indices (*p* < 0.001) and a slightly decreased the Chao1 index, but there were no significant differences (Figure 7A). Meanwhile, 60 mg/kg CAP treatment significantly decreased the Chao1 index compared with the control, while the other groups showed no significant change (Figure 7B). Beta-diversity was measured using PCoA of the weighted UniFrac distance (Figure 7C), revealing distinct bacterial communities between the control and different doses of CAP-treated groups. The results reflected that the gut microbiota of mice in the 40 mg/kg CAP-treated group was significantly different from that in the control group. However, the 60 and 80 mg/kg CAP-treated mice showed no clear changes in intestinal microflora structure from the mice in the control group.

At the phylum level, the dominant phyla in control mice were Bacteroidetes (57.24%) and Firmicutes (37.24%), followed by Deferribacteres (1.68%), Verrucomicrobia (1.41%), and Proteobacteria (1.06%) (Figure 7D). Compared with the control group, 40 mg/kg CAP treatment dramatically reduced the abundance of Bacteroidetes to 34.17% but increased the abundance of Firmicutes (56.64%) (*p* < 0.0001). Notably, CAP treatment at 80 mg/kg significantly reduced the relative abundance of Bacteroidetes, and increased the abundance of Actinobacteria and Proteobacteria (Figure 7E).

**Figure 8:**
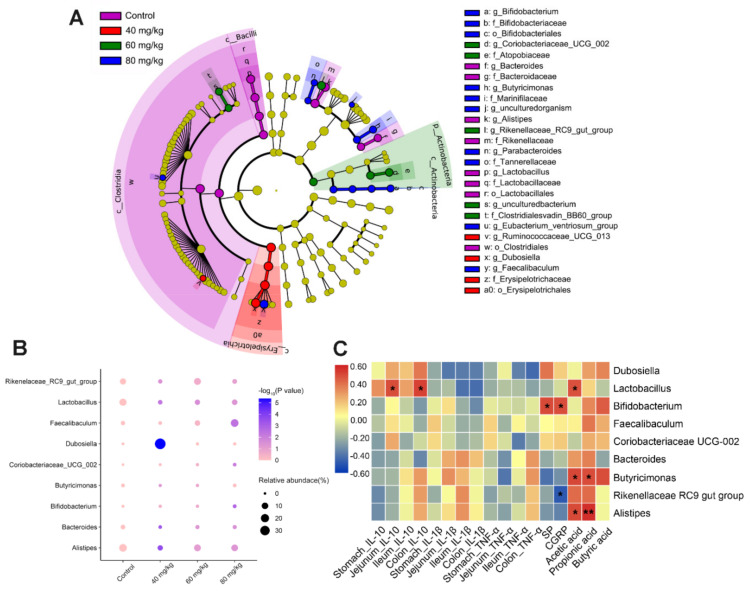
Changes in gut microbiota after CAP treatment. (**A**) LEfSe analysis of gut microbiota is shown as a cladogram among all groups (LDA scores > 3.0). (**B**) Relative abundance of differential microorganisms at the genus level. *p*-values represent the discrepancy compared with the control group. Values are reported after one-way ANOVA followed by the LSD multiple-comparison test. (**C**) Heatmap of Spearman’s correlation between the relative abundance of gut microbiota at the genus level and the relevant indicators. * *p* < 0.05, and ** *p* < 0.01 vs. control.

LEfSe was used to further identify the major bacterial biomarkers that revealed the dominant microorganisms in each group. Dominant bacterial markers of 8, 4, 5, and 10 taxa were found in the control, 40, 60, and 80 mg/kg CAP-treated groups, respectively (Figure 8A). The genera with the greatest differences included *Butyricimonas*, *Lactobacillus*, *Faecalibaculum*, *Coriobacteriaceae_UCG_002*, *Bifidobacterium*, *Rikenellaceae_RC9_gutgroup*, *Bacteroides*, *Alistipes*, and *Dubosiella*. The relative abundance of selected genera with significant differences was analyzed, showing that CAP treatment increased the proportion of *Bifidobacterium* and *Faecalibaculum* in a dose-dependent manner, but only showed a significant change in the 80 mg/kg CAP-treated group (Figure 8B). Moreover, the relative abundance of *Lactobacillus* and *Alistipes* was significantly reduced in the CAP-treated groups, especially in the group treated with 40 mg/kg CAP. Furthermore, compared with the control group, 40 mg/kg CAP treatment drastically elevated the proportion of *Dubosiella*, but reduced the abundance of *Bacteroides*, *Butyricimonas*, and *Rikenellaceae_RC9_gut_group*. Treatment with 80 mg/kg CAP resulted in an increased abundance of *Coriobacteriaceae_UCG_002*.

### 3.7. Spearman’s Correlation Analysis between Gut Microbiota, Cytokines, Neuropeptides, and SCFAs

Spearman’s correlation analysis was performed among the nine main genera and related indicators to investigate the relationships between inflammatory cytokines, gastrointestinal neuropeptides, cecal SCFAs, and gut microbiota (Figure 8C). *Lactobacillus* correlated positively with IL-10 levels in the jejunum and colon (*p* < 0.05) but correlated negatively with pro-inflammatory cytokines (IL-1β and TNF-α). *Bifidobacterium* showed a positive relationship with serum SP and CGRP levels, whereas *Rikenellaceae_RC9_gut_group* showed a negative correlation with serum CGRP levels (*p* < 0.05). In addition, we found that *Butyricimonas* correlated positively with cecal SCFAs levels. *Alistipes* correlated positively with acetic acid and propionic acid levels, whereas *Lactobacillus* only correlated positively with acetic acid levels (*p* < 0.05).

## 4. Discussion

As the main ingredient of peppers, CAP is reported to have a variety of biological characteristics at low doses, such as anti-obesity, pain-relieving, antioxidant, and anti-inflammatory effects. However, when CAP is continuously consumed at high levels, people may experience GI discomforts, such as heartburn, diarrhea, pain, and other symptoms. Therefore, the GI effects of CAP ingestion have received increasing attention. Previous reports have revealed the average CAP consumption in humans, the estimated daily mean CAP intake was 30–150 mg per people, which was 8–37 mg/kg in mice, relatively [17,18]. Recent studies have revealed that oral administration at the dose of 20 mg/kg CAP for 14 days caused obvious inflammatory cell infiltration and cell vacuoles of gastric and jejunal mucosal tissues in rats [7]. Nevertheless, whether CAP affects other GI tissues remains unknown. There is still no clear experimental model and evaluation criteria for the effects of CAP ingestion on GI health. In this study, CAP was administered to mice at 40, 60, and 80 mg/kg to determine the gastrointestinal effects of normal and high CAP consumption.

The results showed that there was no significant damage to stomach tissue and that the damage to intestinal tissues was CAP-dose-related. In particular, compared with the control group, 60 and 80 mg/kg CAP treatment caused a decrease in the V/C ratio, local villus breakage, and shedding in the jejunum and ileum, similar to that shown in a previous report [19]. In addition, we found that oral administration of 80 mg/kg CAP caused inflammatory cell infiltration and significant mucus-producing goblet cell loss in colon tissues, which indicates that CAP induced the injury of GI tissues in a dose–response manner. 

The inflammatory response is closely associated with GI injury. Studies have revealed that the main characteristics of CAP-induced GI inflammation in mice are elevated levels of inflammatory cytokines, especially IL-10, IL-1β, and TNF-α [7]. IL-10 is a key cytokine, which can reduce the release of inflammatory mediators and showed anti-inflammatory properties [20]. IL-1β and TNF-α are essential pro-inflammatory cytokines that cause mucosal inflammation and intestinal barrier damage [21,22]. Our results suggest that 80 mg/kg CAP treatment induces lower levels of IL-10 in all GI tissues and higher levels of IL-1β and TNF-α in the jejunum and colon. These results suggest that high-dose CAP may cause inflammatory injury to the jejunum and colon.

Treatment with CAP could activate the transient receptor potential channel of vanilloid subtype 1 and promote the release of gastrointestinal neuropeptides SP and CGRP, which could act on the central nervous system, regulate the immunoreaction, and are closely related to visceral pain in the GI tract [23]. Our results revealed that oral administration of 40, 60, and 80 mg/kg CAP significantly increased the serum levels of SP and CGRP, which seemed to be CAP-dose-dependent. Studies have shown that CGRP and SP antagonists significantly reduce the inflammatory cells infiltration in the colon of rats [24]. CGRP can induce the migration of T cell and the release of TNF-α by activating mast cells [25], and the blockage of the SP receptor can decrease the inflammation cells in mice with chronic colitis with T cell transfer [26]. Thus, we speculate that the enhanced levels of SP and CGRP in CAP-treated mice may further cause damage to the GI tract, inducing visceral pain.

It is reported that disturbances in the gut microbiota are associated with GI injury. Our results showed the alteration in gut microbiota and its SCFA metabolites after oral administration of different doses of CAP in mice. PCoA analysis revealed that the gut microbiota of 40 mg/kg CAP-treated mice was significantly different from that of the other groups, while the 60 and 80 mg/kg CAP treatments showed no clear changes. At the phylum level, only 40 mg/kg CAP treatment caused an increase in the relative abundance of Firmicutes and a decrease in the abundance of Bacteroidetes compared with the control group, which resulted an increase in Firmicutes/Bacteroidetes ratio. A previous report has revealed that CAP could improve glucose homeostasis in obese diabetic ob/ob mice by significantly increasing the Firmicutes/Bacteroidetes ratio [27]. In addition, 80 mg CAP treatment drastically elevated the relative abundances of Actinobacteria and Proteobacteria, similar to those discussed in previous results [28]. The presence of Proteobacteria is a mark of gut microbiota homeostasis imbalance, closely related to diarrhea symptoms and inflammation [29]. 

By identifying the major bacterial markers at the genus level, we found that the increase in the abundance of Actinobacteria was primarily attributed to the increased abundance of *Bifidobacterium*. The relative abundance of *Bifidobacterium* was dose-related to CAP, and the correlation analysis showed that the abundance of *Bifidobacterium* was also positively related to the serum levels of SP and CGRP. The local release of neuropeptides can alter the composition of the gut microbiota by regulating the immune response in the GI tract, whereas bacteria can also sense neuropeptides through membrane proteins [30,31]. Thus, we speculate that the dose-dependent increase in serum SP and CGRP in CAP-treated mice may be related to the promotion of the relative abundance of *Bifidobacterium*. Additionally, the 60 and 80 mg/kg CAP-treated groups significantly enriched the abundance of *Faecalibacterium*, similarly to previous results [32]. *Faecalibacterium* is considered a bioindicator of GI disorders and displayed a positive correlation with butyric acid/SCFA production [33,34]. This may be the reason for the significantly high level of cecal butyric acid in 60 and 80 mg/kg CAP-treated mice. 

Moreover, the abundance of *Lactobacillus*, *Bacteroides*, and *Alistipes* was reduced in the CAP-treated groups. *Lactobacillus* belongs to the phylum Firmicutes, possessing the capacity to inhibit pathogens or relieve inflammation [35]. Our results showed that the abundance of *Lactobacillus* positively related to IL-10 levels in the jejunum and colon (*p* < 0.05), suggesting that CAP may influence the anti-inflammatory properties of GI tissues by decreasing the abundance of *Lactobacillus* in the intestinal microflora. The decreased abundance of *Bacteroides* after CAP treatment has been demonstrated in previous studies by [27,36]. Furthermore, *Butyricimonas* is known for its ability to produce butyric acid [37]. The abundance of *Butyricimonas* was decreased in the 40 and 60 mg/kg CAP groups, but significantly increased in the 80 mg/kg group, suggesting that high butyric acid levels in 80 mg/kg CAP-treated mice may be due to the promotion of *Butyricimonas* growth. 

Notably, compared with the control group, 40 mg/kg CAP treatment drastically elevated the proportion of *Dubosiella*, and 80 mg/kg CAP treatment resulted in an increased abundance of *Coriobacteriaceae_UCG_002*. *Dubosiella* was reported to inhibit obesity in mice in previous studies [38], while *Coriobacteriaceae_UCG_002* was beneficial to the host by producing the essential amino acids and fermenting dietary proteins. These surprising findings required more follow-up research [39].

## 5. Conclusions

In summary, mice were intragastrically administered with CAP at three doses to evaluate the effects of CAP on GI health. The results showed that administration of 40 mg/kg CAP did not have significant negative effects on the GI tract in mice, while 60 and 80 mg/kg CAP caused GI injury by damaging GI tissues and decreasing the levels of anti-inflammatory cytokines (IL-10). Inflammation and histopathological changes were significant in the jejunum, ileum, and colon, but only slight in the stomach. CAP increased serum SP and CGRP levels in a dose-dependent manner, which may induce an immune response and visceral pain. The levels of cecal SCFAs also significantly changed in the 80 mg/kg CAP-treated groups. These effects of CAP might be related to the regulation of gut microbiota, especially *Bifidobacterium, Lactobacillus, Faecalibacterium*, and *Butyricimonas*. Moreover, the underlying mechanism of the correlation between serum neuropeptides and specific gut microbiota needs to be studied, suggesting that probiotics, as members of the gut microbiota, may be an alternative in relieving CAP-induced GI injury. These data will reveal the effects of CAP on GI health, provide insight into the experimental model of CAP-induced GI injury, and enrich the correlation analysis between CAP ingestion and gut microbiota.

## Figures and Tables

**Table 1:** Animal experimental design.

Group	Daily Gavage Treatment	Volume	Sample Size
Control	Vehicle	200 μL	*n* = 6
40 mg/kg	CAP at a concentration of 40 mg/kg bodyweight	200 μL	*n* = 6
60 mg/kg	CAP at a concentration of 60 mg/kg bodyweight	200 μL	*n* = 6
80 mg/kg	CAP at a concentration of 80 mg/kg bodyweight	200 μL	*n* = 6

## Data Availability

All data presented in this study are available in the main body of the manuscript.

## Supplementary Materials

The following are available online at https://www.mdpi.com/article/10.3390/foods11050686/s1, Figure S1. Effects of CAP on daily food intake and water intake of each mice: (A) Food intake, (B) Water intake; Figure S2. Assessment of colonic tissues: (A) Height of colonic crypts, (B) Goblet numbers per crypt.

## Abbreviations

ANOVA	Analysis of variance
CAP	Capsaicin
CGRP	Calcitonin gene-related peptide
GC-MS	Gas chromatograph-mass spectrometry
GI	Gastrointestinal
H&E	Hematoxylin and eosin
LDA	Linear discriminant analysis
LEfSe	Linear discriminant analysis effect size
OTU	Operational taxonomic unit
PCoA	Principal coordinates analysis
SCFAs	Short-chain fatty acids
SEM	Standard error of the mean
SP	Substance P
