# Supplementary material for: Capsaicin, the Spicy Ingredient of Chili Peppers: Effects on Gastrointestinal Tract and Composition of Gut Microbiota at Various Dosages
Source: Foods. 2022 Feb 25;11(5):686. doi: 10.3390/foods11050686 (PMC8909049; doi:10.3390/foods11050686)
Supplement: Supplementary file 1 [file foods-11-00686-s001.zip › foods-1578322-supplementary.pdf]

Supplementary Material

Supplementary Material 1:

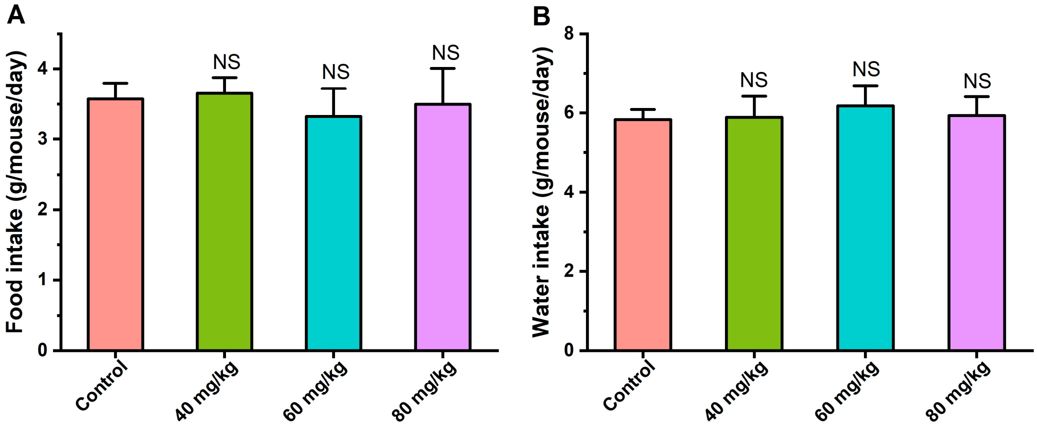

Figure S1. Effects of CAP on daily food intake and water intake of each mice: (**A**) Food intake, (**B**) Water intake. NS: not significant.

Supplementary Material 2:

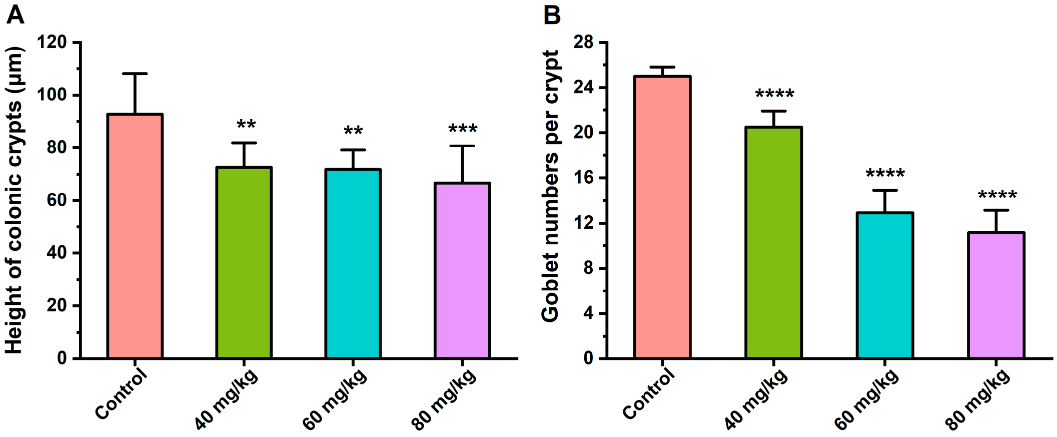

Figure S2. Assessment of colonic tissues: (**A**) Height of colonic crypts, (**B**) Goblet numbers per crypt. ** $p < 0.01$, *** $p < 0.001$ and **** $p < 0.0001$ vs. control.
